# *Arabidopsis thaliana* genes with codon usage bias similar to that of *B. amyloliquefaciens* are involved in the regulation of *A. thaliana* adaptation to high calcium stress by *B. amyloliquefaciens*

**DOI:** 10.3389/fpls.2025.1623360

**Published:** 2025-09-01

**Authors:** Fei Li, Qinye Zhang, Yuntong Lu, Xiaoyan Chen, Xing Liu, Xiangting Qiu, Yunying Gu, Puchang Wang, Jie Liu

**Affiliations:** ^1^ School of Life Sciences, Guizhou Normal University, Guiyang, Guizhou, China; ^2^ Key Laboratory for Information System of Mountainous Area and Protection of Ecological Environment of Guizhou Province, Guizhou Normal University, Guiyang, Guizhou, China; ^3^ State Key Laboratory of Microbial Technology, Shandong University, Qingdao, Shandong, China

**Keywords:** plant-endophyte interaction, codon usage patterns, host adaptation, *A. thaliana*, *B. amyloliquefaciens*

## Abstract

**Introduction:**

Codon usage bias (CUB) can influence host-microbe interactions and stress adaptation. In this study, we aimed to investigate how codon usage bias (CUB) similarity between *Arabidopsis thaliana* and *Bacillus amyloliquefaciens* influences their interaction and contributes to the adaptation of *A. thaliana* to high calcium stress.

**Methods:**

The CUB indices of both species were computed, and genes with high correlations were identified. The transcriptome sequencing data of gene expression in *A. thaliana* cultured under normal and high calcium conditions, with and without *B. amyloliquefaciens* treatment was used to analyze the expression of *A. thaliana* genes with CUB similar to that of B. amyloliquefaciens in relation with the adaptation of *A. thaliana* to high calcium stress and the interaction between both organisms.

**Results:**

We identified 19210 *A. thaliana* genes with CUB similar to *B. amyloliquefaciens* and 95 *B. amyloliquefaciens*-responsive and calcium-responsive genes in *A. thaliana*, which were involved in transport, carbohydrate metabolism, and response to chemical, and cellular homeostasis. Differential expression analysis showed a total of 733 A. thaliana genes with CUB similar to *B. amyloliquefaciens* to be dysregulated, among which 47 changed when *A. thaliana* was cultivated in the presence of the *B. amyloliquefaciens* LZ04 strain, 643 under high calcium condition and 43 with calcium treatment and the presence of the *B. amyloliquefaciens* LZO4 strain. The gene ontology (GO) biological processes termed among others of response to endogenous stimulus, response to oxygen containing compound, response to organic substance, response to abiotic and biotic stimuli, response to stress, and response to light stimulus, regulation of hormone levels, response to nutrient levels, post-embryonic plant morphogenesis, metabolic process, cell growth.

**Discussion:**

These findings highlight the importance of CUB in the interaction between *A. thaliana* and *B. amyloliquefaciens* as well as in the adaptation of *A. thaliana* to high calcium stress. They also show the underlying regulatory role of *B. amyloliquefaciens*, which could help develop new tactics for improving *A. thaliana* growth and yield in karst regions. A more elaborate analysis of the value of CUB in the interaction of these two organisms could assist in engineering host- sensitive micro-organism strains and enhance the microbial-based approaches for the improvement of *A. thaliana* growth and yield in such areas and for managing abiotic stress in crops.

## Introduction

*Arabidopsis thaliana* is an annual dicotyledonous, tiny flowering plant in the mustard family with a less upright growth pattern than most crops of interest in agriculture. Important variation can be observed in the gene families of *A. thaliana* and plant species with large genome complexity, such as rice (*Oryza sativa*) (Nelson et al., 2004; Lawson and Zhang, 2006). Despite these differences, *A. thaliana* is considered a model system for plant biology studies due to its fully mapped genome, small genome size, genome duplication, polyploidization, and rapid growth cycle (Goodman et al., 1995; Meinke et al., 1998). Knowledge of *A. thaliana* biology can therefore help to enhance the breeding of dicotyledon crops as well as monocot crops such as rice (*O. sativa* L.), bread wheat (*Triticum aestivum* L.), and maize (*Zea mays* L.).

Plant-growth-promoting rhizobacteria (PGPR) are microbes in the vicinity of roots that positively impact the growth and health of host plants and help these plants tolerate stress and fight against diseases (Zhao et al., 2015; Chen et al., 2017; Eckshtain-Levi et al., 2020). Plants growing in harsh conditions with salt, drought, thermal, and heavy metal stresses rely on symbiotic microorganisms, including PGPR, for adaptation. In our previous study, we analyzed bacterial communities in soil with high calcium content and in the roots and leaves of *Cochlearia henryi*, another species with genetics closely related to *A. thaliana*, using high-throughput amplicon sequencing (Li et al., 2018). The results showed that *C. henryi* selectively co-exists with specific bacteria, indicating its adaptation to high calcium stress and the importance of bacterial communities in this adaptation (Li et al., 2018). The interaction of *A. thaliana* with PGPR has been documented and involves a complex interplay between both organisms. For example, it was demonstrated that *B. amyloliquefaciens* FZB42 volatiles induce salt tolerance in *A. thaliana* through the jasmonic acid signaling pathway (Liu et al., 2020). Moreover, we have demonstrated in previous studies that *B. subtilis* and *A. thaliana* form a model interaction system for studying the role of volatile organic compounds in the interchange between plants and bacteria (Li et al., 2019). Additionally, *A. thaliana* interacts strongly with PGPR, especially *B. subtilis*, attracting it to the roots through chemotaxis using chemoreceptors (Allard-Massicotte et al., 2016). The colonization process starts with the germination of *B. subtilis* spores at the root-soil interface, followed by a brief vegetative period before reverting to spores (Charron-Lamoureux and Beauregard, 2019). In another study, we found that *B. amyloliquefaciens* PDR1 from the root of karst adaptive plants enhances the resistance of *A. thaliana* to alkaline stress via regulating the activity of plasma membrane H (+)-ATPase (Li et al., 2020c). Furthermore, we demonstrated that *B. amyloliquefaciens* LZ04 improves the resistance of *A. thaliana* to high calcium stress, potentially through a lncRNA-miRNA-mRNA regulatory network (Li et al., 2020b). In an additional study, we discovered that treating *A. thaliana* with *B. amyloliquefaciens* LZ04 can improve its resistance to high calcium stress by regulating certain genes in calcium-related gene families (Gong et al., 2021). Apart from our studies, other scholars conducted transcriptome profiling to understand the mechanisms involved in the interaction between *B. amyloliquefaciens* FZB42 and *A. thaliana* under induced systemic salt tolerance (Liu et al., 2017). However, attempts at extending the knowledge of the molecular basis of the crosstalk between *A. thaliana* and PGPR are very important in the practice of breeding. This necessitates the exploration of molecular mechanisms of the interaction in all aspects.

Codon usage bias (CUB) refers to the preferential use of certain codons to encode the same amino acid (Jiang et al., 2022). The plant-bacteria interaction can potentially be affected by CUB, which can impact the expression levels of genes involved in this interaction (Qin et al., 2022). Experimental evolution to develop isolates with improved capability to form root biofilm in colonized root suggests a possible role of evolution, and thus, CUB in this colonization process (Blake et al., 2021a, b). A previous study found that organisms capable of inhabiting multiple environments, such as facultative organisms, mesophilic, and pathogenic bacteria, have lower translational efficiency, which suggests the role of CUB in their need to adapt to different environments (Arella et al., 2021). Therefore, PGPR may exhibit codon usage patterns that are similar to those of their plant hosts, and hence facilitating efficient communication between the two organisms. Additionally, CUB could impact translation efficiency (Nambou and Anakpa, 2020; Nambou et al., 2022), which could ultimately affect protein expression levels in both the plant and PGPR. Despite many studies on plant-PGPR interactions, there is little research on the role of CUB in this interaction. As well, very few studies have focused on the interaction between *A. thaliana* and *B. amyloliquefaciens*. More research is therefore needed to understand how CUB impacts their interaction, which may help improve crop yields and plant health.

The aim of this study was to determine whether there is a correlation between the CUB of *A. thaliana* and *B. amyloliquefaciens* and its relevance to the adaptation of *A. thaliana* to high calcium stress. Specifically, we sought to compare the CUB indices of both species to explore the roles of *A. thaliana* genes that exhibit a CUB similar to that of *B. amyloliquefaciens* in their interaction. This was investigated using available transcriptome data from plants grown under high calcium conditions, both with and without *B. amyloliquefaciens*. We hypothesize that this research will enhance our understanding of how *B. amyloliquefaciens* influences the growth and yield of *A. thaliana*. This understanding will serve as a preliminary step toward developing microbial-based strategies to increase *A. thaliana* yield in karst areas where the soil has a high calcium content.

## Methods

### Sequence download

To acquire the genome coding sequence (CDS) data of both *A. thaliana* and *B. amyloliquefaciens*, we searched the NCBI (National Center for Biotechnology Information) database and downloaded the CDS sequences in FASTA format.

### Computation of codon usage indexes

The VHCUB library in R was used to compute various CUB variables. These variables included the nucleotide content (GC, GC1, GC2, and GC3), the Enc, CAI, siD, SCUO, and RCDI. This package was also used to visualize the PR2 plot and the Enc-GC3 plot. The ComplexHeatmap package in R was used to visualize the density heatmap of the nucleotide content, while the density was calculated using the ‘stats’ package in R and plotted using the “plot” function in R. To analyze the correlation between different CUB indexes, we employed the Hmisc package in R.

### RSCU-based correlation analysis between *A. thaliana* and *B. amyloliquefaciens*

To analyze the correlation between the RSCU values of *A. thaliana* genes and *B. amyloliquefaciens* genes, we first calculated the RSCU values for each gene in both organisms. We then merged the RSCU values of CDS sequences from both organisms into an RSCU value table. Next, the Hmisc package in R was employed to compute the correlation between the genes of both organisms based on the RSCU values. The *A. thaliana* genes with a correlation coefficient r>=0.5 and p<0.05 were considered as those *A. thaliana* genes with CUB similar to that of *B. amyloliquefaciens*. The choice of r >= 0.5 was based on previous studies (Nambou and Anakpa, 2020; Nambou et al., 2022); this cutoff can provide a balance among sensitivity and specificity in the identification of *A. thaliana* genes with CUB similar to *B. amyloliquefaciens*. Indeed, moderate positive and significant correlations in biological traits, such as codon usage, may be detected at a median cutoff of 0.5; using higher thresholds, like r > 0.7, might exclude meaningful genes, limiting the analysis and potentially missing significant genetic links. In the same vein, false discovery rate (FDR) p-values were not considered in the computation of Pearson correlation to avoid being overly stringent, but p < 0.05 was considered to ensure the selected r values reflected both statistically significant and biologically relevant correlation among genes.

### Differential expression analysis of *A. thaliana* genes with CUB similar to that of *B. amyloliquefaciens* in the roots of *A. thaliana* under calcium-stress conditions in combination with or without *B. amyloliquefaciens* treatment

In our previous study, we demonstrated that treatment with *B. amyloliquefaciens* LZ04 enhanced the resistance of *A. thaliana* under high calcium stress (Gong et al., 2021). The culture conditions and the indexes measured were as described previously (Gong et al., 2021). Briefly, the *B. amyloliquefaciens* LZ04 strain was grown on LB agar plates at 28°C, while seeds of the *A. thaliana* ecotype Columbia were germinated on 0.6% MS medium after sterilization. In the experiments, separate plates were used for each setup. Four treatment groups were established: a control group with *A. thaliana* grown without CaCl_2_, a group with LZ04 and no CaCl_2_, a group with 40 mM CaCl_2_, and a group with both 40 mM CaCl_2_ and LZ04. After planting, the plates were kept at 23°C for 48 hours. *E. coli* served as a control strain to verify the specific effects of *B. amyloliquefaciens* LZ04. Then, root tissues from *A. thaliana* subjected to each treatment were collected for transcriptome analysis. Total RNA was isolated from these root samples (3 samples by group) using the Plant RNA Purification Reagent (Invitrogen), and strand-specific sequencing was performed on an Illumina HiSeq 4000 platform. The generated transcriptome data by RNA-sequencing was deposited in the China National GeneBank DataBase (CNGBdb) under the project accession numbers CNP0000745 and CNP0000640 (Gong et al., 2021). Herein, we extracted the count table corresponding to *A. thaliana* genes with CUB similar to that of *B. amyloliquefaciens*. Differential expression analysis was achieved employing the DESeq2 and edgeR packages developed using R programming to identify DEGs based on the extracted RNA-seq expression count table. In the analysis employing edgeR, the count data were transformed into a DGEList object, and subsequently normalized with the TMM (trimmed mean of M-values) method. The genes with low expression levels were screened out using the filterByExpr function. A quasi-likelihood negative binomial generalized log-linear model was fitted using glmQLFit, and differential expression was evaluated using the glmQLFTest function, with genes categorized as differentially expressed based on an adjusted p-value cutoff of <0.05 and a fold change threshold of >1. In the analysis employing DESeq2, raw counts were imported into a DESeqDataSet object, followed by the removal of low-count genes. After normalization, DEGs were identified using the Wald test with significance determined by an adjusted p-value (padj) <0.05. Subsequent to the analysis, the upregulated as well as the downregulated DEGs obtained from both analysis methods were merged to obtain the common genes for further analysis. Merging DEGs from DESeq2 and edgeR could improve the statistical power, validate results, provide a comprehensive view of gene expression alteration, and improve the filtration of noise, leading to more robust biological insights. We utilized the R “pheatmap” package (https://cran.r-project.org/web/packages/pheatmap/index.html) to visualize the heatmap of DEGs.

### Cluster analysis of the expression profiles of *A. thaliana* genes with CUB similar to that of *B. amyloliquefaciens*

We used the Short Time-series Expression Miner software (Ernst and Bar-Joseph, 2006) to conduct a cluster analysis of *A. thaliana* genes with CUB similar to that of *B. amyloliquefaciens*.

### Protein-protein interaction network

The protein-protein interaction (PPI) networks of sets of genes were generated by using the string database (https://string-db.org/), setting the confidence threshold at 0.150. The generated networks were downloaded and subsequently visualized in Cytoscape software. The MCODE plugin was used for detecting hub genes and hub clusters.

### GO enrichment analysis

To explore the biological functions of these genes, we performed GO analysis using the TBtools software. The resulting most significant terms of interest for biological process, cellular component, and molecular function were visualized. Terms with p value<0.05 were considered significant.

### Quantitative real-time PCR

RT-qPCR was utilized to confirm the transcriptome data by checking the expression of selected genes in the roots of *A. thaliana* cultivated under the above-mentioned conditions. Total RNA was extracted from the root tissues with the RNA simple Total RNA extraction kit (Tiangen Biotech-Beijing Co., Limited, Beijing, China) in accordance with the manufacturer’s procedure. To eliminate genomic DNA, DNase (DNAfree kit from Ambion) treatment was performed. Afterwards, the quality and quantity of RNA were determined by measuring the absorbance with NanoDrop and performing agarose gel electrophoresis. Successful removal of gDNA was confirmed by running no-reverse-transcriptase (No-RT) control reactions in qPCR; the absence of amplification in these No-RT samples indicated minimal or no gDNA contamination (Laurell et al., 2012; Padhi et al., 2016). The extracted DNase−treated RNA was reverse transcribed into cDNA using PrimeScript^®^ II First Strand cDNA Synthesis Kit (TaKaRa, Tokyo, Japan). The ensuing cDNA was used as a template for qRT-PCR. The primers used were as summarized in **Supplementary Table S1**. The qRT-PCR was performed on StepOnePlus Real-Time PCR System (Applied Biosystems - Roche Molecular Systems Inc., Branchburg, NJ) using SYBR PrimeScript^®^ RT-PCR Kit (TaKaRa, Tokyo, Japan). The target gene relative expression levels were normalized to the housekeeping gene GAPDH with the 2^–ΔΔCt^ method. The experiment was performed in triplicate from each of three independent biological replicates.

### Statistical analysis

Statistical analysis was performed using GraphPad Prism 8 (GraphPad Software, San Diego, California, USA). The data was expressed as mean ± SD (standard deviation). A one-way ANOVA, followed by Tukey’s *post hoc* multiple comparison test, was performed to determine the significance of differences among the groups, using a p-value cutoff of 0.05.

## Results

### Nucleotide composition analysis

**Figure 1:**
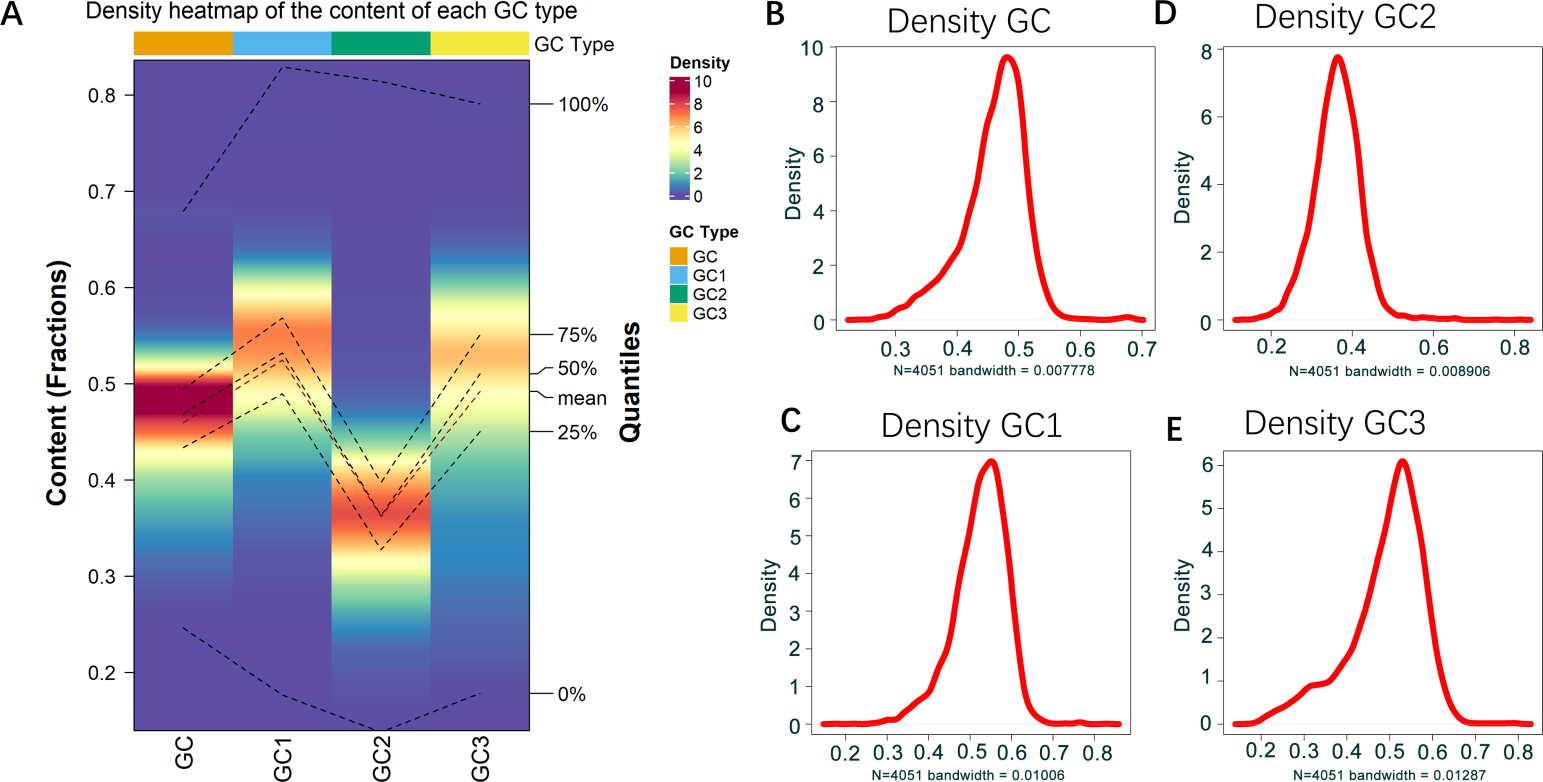
GC content distribution in *B. amyloliquefaciens* genome. **(A)** Density heatmap showing the distribution of GC, GC1, GC2, and GC3 in *B. amyloliquefaciens* genome. **(B)** Density plot showing the distribution of GC in *B. amyloliquefaciens* genome. **(C)** Density plot showing the distribution of GC1 in *B. amyloliquefaciens* genome. **(D)** Density plot showing the distribution of GC2 in *B. amyloliquefaciens* genome. **(E)** Density plot showing the distribution of GC3 in *B. amyloliquefaciens* genome.

To get insights on the distribution of GC (guanine and cytosine bases), GC1 (GC at the first codon position), GC2 (GC at the second codon position), and GC3 (GC at the third codon position) content in the genome of *B. amyloliquefaciens*, these variables were calculated and used for density analysis. The density heatmap and density plot of each variable were as indicated in **Figure 1**. The results showed that the lowest value for GC content in *B. amyloliquefaciens* genes was 0.2228, while the maximum GC content recorded was 0.7023 (**Figures 1A, B**). The minimum density value of GC content observations was 0.000147, while the mean density value was 2.083060 (**Figures 1A, B**). The maximum density of GC was 9.628902 (**Figures 1A, B**). Moreover, the content in GC1 ranged from 0.1463 to 0.8591, while the density ranged from 0.000113 to 6.978296 (**Figures 1A, C**). The median GC1 content was 0.5027 (**Figures 1A, C**), while GC2 content values were between 0.1112 and 0.8407, with a peak around 0.4760 (**Figures 1A, D**). The average density of GC2 content observations was 1.36942 (**Figures 1A, D**). GC3 content was from 0.1395 to 0.8293, with a density peak around 0.4844 and mean density value of 1.448239 (**Figures 1A, E**).

### Effective number of codons of the coding sequences and codon usage adaptation of *B. amyloliquefaciens* to *A. thaliana*

**Figure 2:**
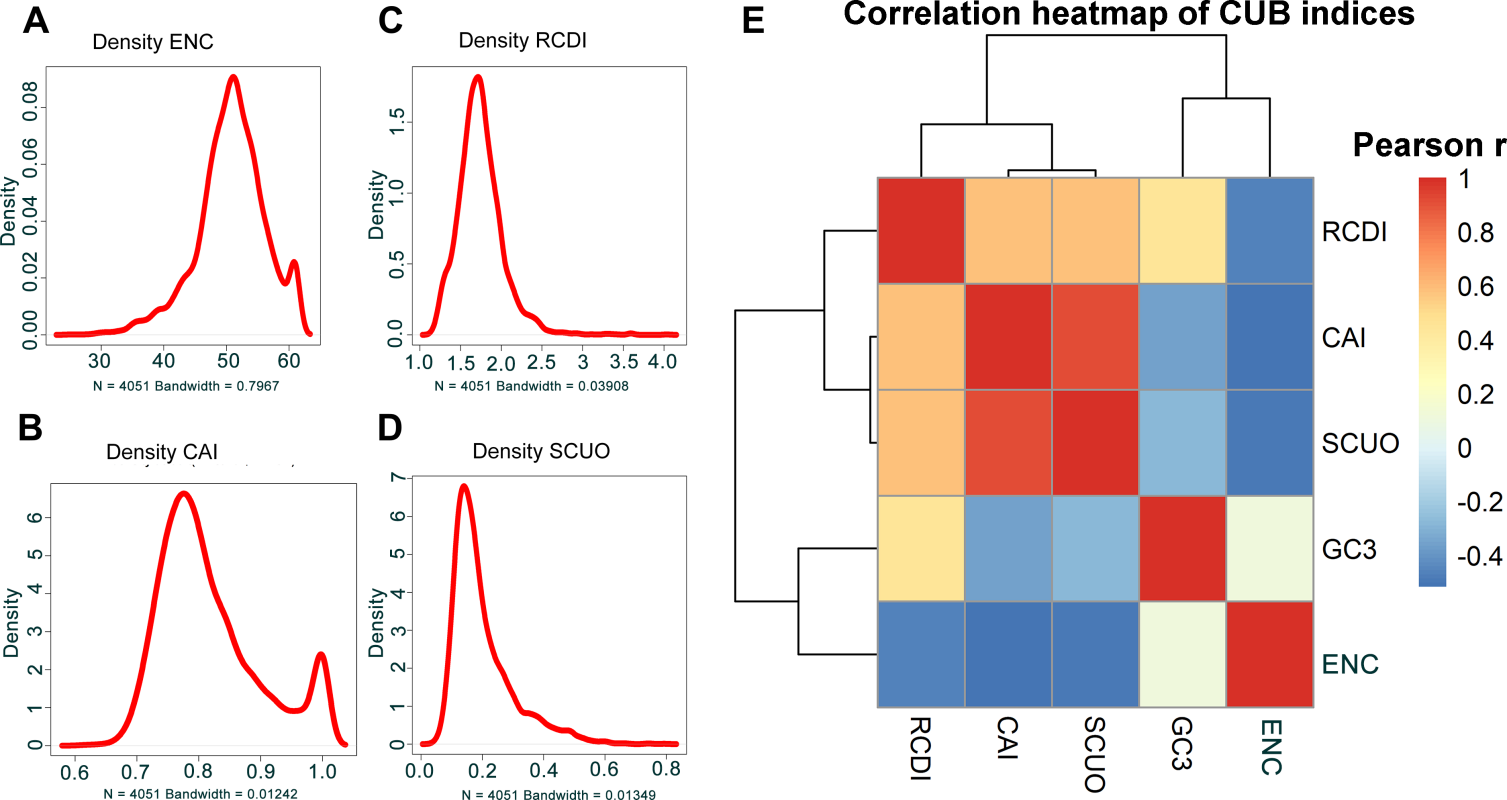
ENC of the coding sequences and indexes codon usage adaptation of *B. amyloliquefaciens* to *A. thaliana*. **(A)** Density distribution of the ENC values of the coding sequences of *B. amyloliquefaciens*. **(B)** Density distribution of the CAI values of the coding sequences of *B. amyloliquefaciens* relative to *A. thaliana*. **(C)** Density distribution of the RCDI values of the coding sequences of *B. amyloliquefaciens* relative to *A. thaliana*. **(D)** Density distribution of the SCUO values of the coding sequences of *B. amyloliquefaciens* relative to *A. thaliana*. **(E)** Pearson correlation analysis of the correlations among Enc, GC3, SCUO, CAI, and RCDI values of the coding sequences of *B. amyloliquefaciens*.

For the description of the CUB of the coding sequences of *B. amyloliquefaciens*, the Effective Number of Codons (ENC), a measure utilized in order to determine the codon preference bias in a gene or in any genome, was computed. A smaller ENC value indicates a stronger CUB. The distribution of ENC values of the coding sequences is displayed in **Figure 2A**. The ENC values were distributed from 25.20 to 61.00, with a mean ENC value of 50.75 (**Figure 2A**).

To investigate the adaptability and optimization of codon usage in *B. amyloliquefaciens* and its host, *A. thaliana*, we employed various measures, including CAI (Codon Adaptation Index), RCDI (Relative Codon Deoptimization Index), and SiD (Similarity Index). We found that *B. amyloliquefaciens* had a mean CAI value of 0.8224, which ranged from 0.6163 to 1 (**Figure 2B**; **Additional File 1**). The density plot indicated that most of the sequences in *B. amyloliquefaciens* had a CAI value between 0.7 and 1 (**Figure 2B**; **Additional File 1**). The RCDI values varied from 1.148 to 4.037, and the mean RCDI was 1.745 (**Figure 2C**; **Additional File 1**). In addition, the SCUO (Synonymous Codon Usage Order) values were between 0.04468 and 0.79292, with the average SCUO was 0.20028 (**Figure 2D**; **Additional File 1**). Furthermore, the results showed that the SiD value was 0.4921. In addition, a negative correlation was recorded between the CAI values and the ENC and GC3 values of *B. amyloliquefaciens* (**Figure 2E**; **Additional File 1**). Additionally, positive correlations were found among the CAI, RCDI, and SCUO values (**Figure 2E**; **Additional File 1**).

### ENC-plot analysis and parity analysis

**Figure 3:**
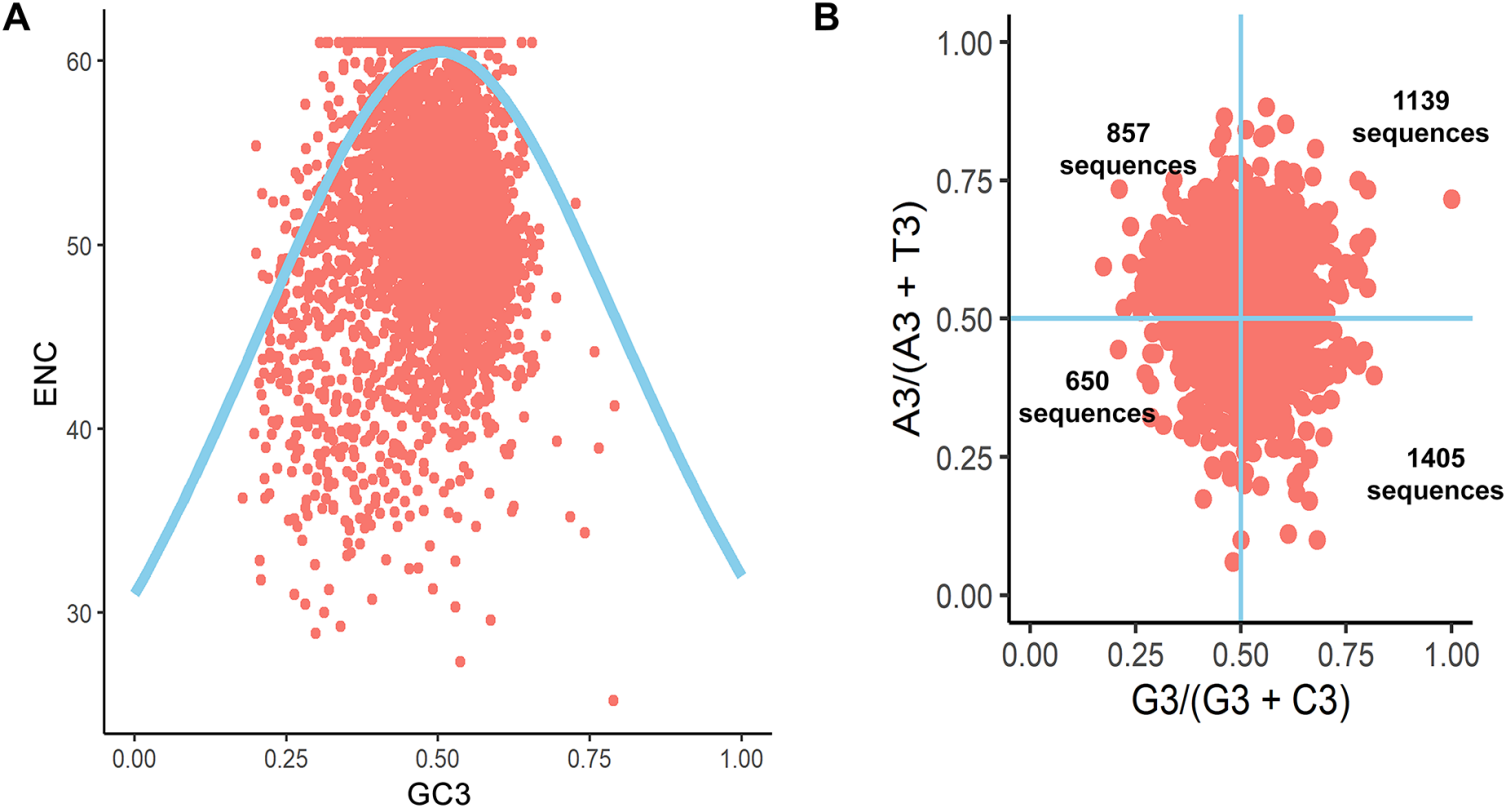
ENC plot and Parity analysis of the coding sequences of *B. amyloliquefaciens*. **(A)** ENC plot. **(B)** Parity plot.

To investigate the factors influencing the codon usage of *B. amyloliquefaciens*, an ENC plot was created. If codon bias is solely influenced by natural selection pressure, all points would lie below the expected ENC curve. However, points above the curve suggest that the codon usage of *B. amyloliquefaciens* is influenced by mutation pressure. As shown in **Figure 3A**, the ENC values of the coding sequences of *B. amyloliquefaciens* were found to be distributed on both sides of the expected ENC curve.

To investigate CUB in the coding sequences of *B. amyloliquefaciens*, we employed the parity rule 2 (PR2) bias plot. This tool allows for analysis of whether natural selection and mutation are at play. If there is no deviation from these factors, the points on the graph will appear in the middle, located at coordinates (0.5, 0.5). In **Figure 3B**, it can be observed that the majority of points on the PR2 plots for *B. amyloliquefaciens* are located in the bottom right and upper right quadrants. There were 1,139 sequences found in the top right quadrant, which suggests that these sequences contain A- and/or U-ending codons (**Figure 3B**). The group in the bottom right was 1,405 sequences with G- and C-ending codons (**Figure 3B**).

### A- and T-ending codons are preferred in coding sequences of *A. thaliana* and *B. amyloliquefaciens*

The RSCU (Relative Synonymous Codon Usage) values were computed to examine the codon usage patterns of the coding sequences of *A. thaliana* and *B. amyloliquefaciens*. As shown in **Table 1**, we observed that the coding sequences of *A. thaliana* and *B. amyloliquefaciens* used the codons ending with A or T preferentially. In total, 14 codons having A or T endings were identified to be preferred by both organisms. These codons were those coding for amino acids such as Arginine, Asparagine, Aspartic acid, Glutamic acid, Glutamine, Glycine, Histidine, Isoleucine, Leucine, Phenylalanine, Serine, Threonine, and Tyrosine (**Table 1**).

**Table 1:** RSCU values of codons in *A. thaliana* and *B. amyloliquefaciens.*.

Amino acid	Codon	*A. thaliana*	*B. amyloliquefaciens*
Alanine	GCA	1.2151	0.8734
	GCC	0.73	1.0437
	GCG	0.5492	1.2559
	GCT	1.467	0.8102
Arginine	**AGA**	**2.312**	**1.4859**
	AGG	1.1179	0.5551
	CGA	0.8347	0.4082
	CGC	0.4115	1.286
	CGG	0.5674	1.236
	CGT	0.7338	0.9398
Asparagine	AAC	0.877	0.9128
	**AAT**	**1.11**	**1.056**
Aspartic acid	GAC	0.6779	0.7853
	**GAT**	**1.299**	**1.17**
Cysteine	TGC	0.7462	0.87
	TGT	1.223	0.6289
Glutamic acid	**GAA**	**1.137**	**1.386**
	GAG	0.8462	0.5866
Glutamine	**CAA**	1.272	0.8818
	CAG	0.7032	1.074
Glycine	**GGA**	**1.492**	**1.2316**
	**GGC**	0.6681	1.406
	GGG	0.6759	0.671
	GGT	1.132	0.6638
Histidine	CAC	0.7728	0.6628
	**CAT**	**1.195**	**1.207**
Isoleucine	ATA	0.8348	0.3984
	ATC	0.9619	1.219
	**ATT**	**1.1965**	**1.363**
Leucine	CTA	0.6531	0.1604
	CTC	0.9769	0.7984
	CTG	0.6783	1.682
	**CTT**	**1.3293**	**1.351**
	TTA	1.013	1.1349
	TTG	1.346	0.8572
Lysine	AAA	1.1216	1.444
	AAG	0.8696	0.5441
Methionine	ATG	0.9756	0.9906
Phenylalanine	TTC	0.8347	0.711
	**TTT**	**1.157**	**1.257**
Proline	CCA	1.423	0.3535
	CCC	0.6148	0.5268
	CCG	0.7237	2.108
	CCT	1.202	0.8768
Serine	AGC	0.8055	1.3918
	AGT	0.9304	0.5893
	**TCA**	**1.2735**	**1.3601**
	TCC	0.8038	0.9237
	TCG	0.6166	0.6532
	**TCT**	**1.565**	**1.0463**
Threonine	**ACA**	**1.364**	**1.247**
	ACC	0.8273	0.7484
	ACG	0.6136	1.442
	ACT	1.1797	0.5207
Tryptophan	TGG	0.952	0.781
Tyrosine	TAC	0.7692	0.749
	**TAT**	**1.203**	**1.199**
Valine	GTA	0.7533	0.7099
	GTC	0.7817	1.215
	GTG	0.9508	1.0915
	GTT	1.499	0.9599

Codons highlighted in bold were identified to be preferred by both organisms.

### Identification of *A. thaliana* genes with CUB similar to *B. amyloliquefaciens* CUB, their regulatory network, and functional roles

**Figure 4:**
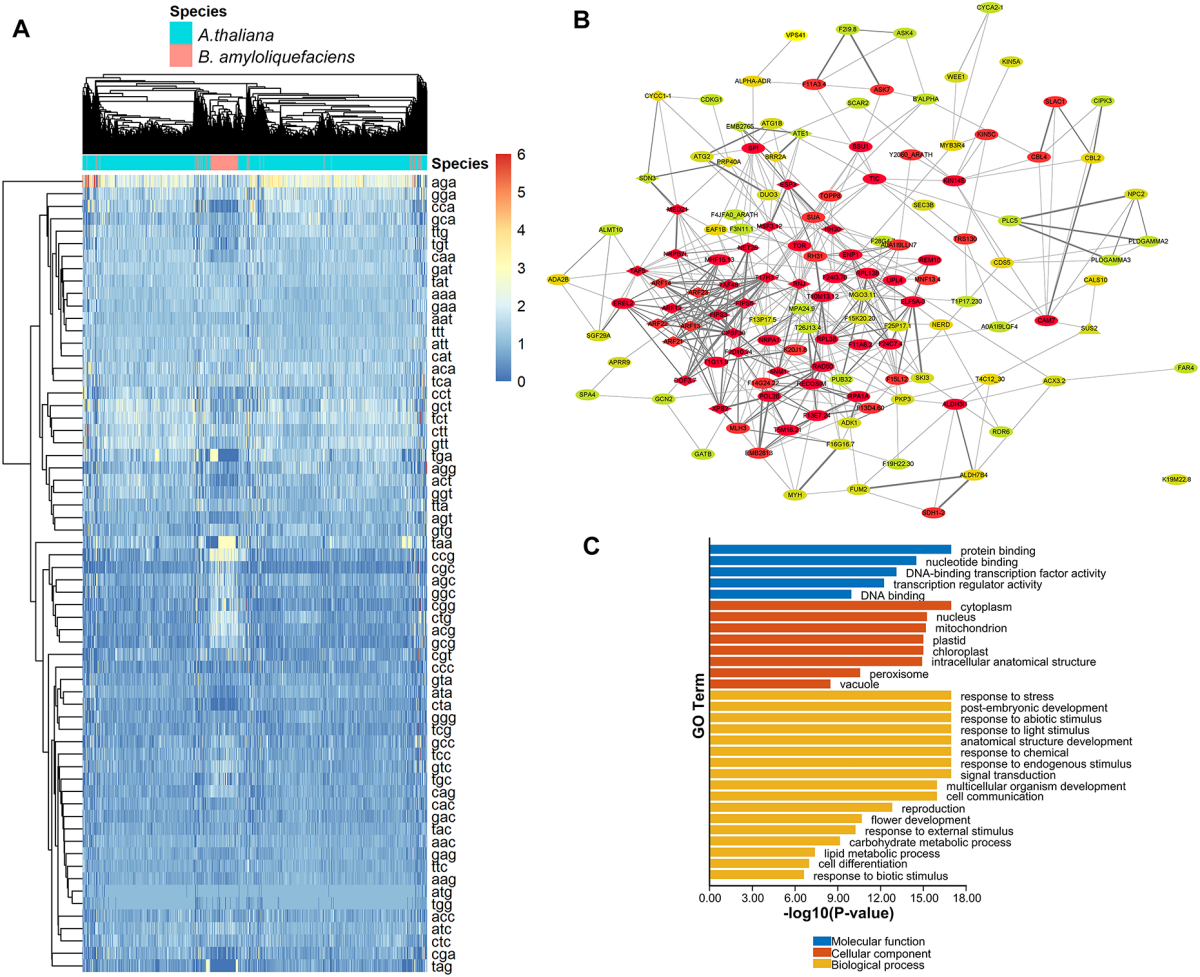
Identification of *A. thaliana* genes with CUB similar to *B. amyloliquefaciens* CUB, their regulatory network, and functional roles. **(A)** Heatmap showing the RSCU values of each codon in the coding sequences of *A. thaliana* genes and *B. amyloliquefaciens* with significant correlations. **(B)** Hub clusters of the PPI network of the most relevant 2000 genes of *A. thaliana* genes with CUB similar to *B. amyloliquefaciens* CUB. Proteins of the same shape belong to the same cluster while the color indicates the degree obtained from network analysis (higher degrees are represented by red, yellow indicates medium degree, while green represents low degree). **(C)** Functional enrichment of *A. thaliana* genes with CUB similar to *B. amyloliquefaciens* CUB.

In order to identify *A. thaliana* genes with CUB similar to that of *B. amyloliquefaciens*, Pearson correlation analysis based on RSCU values was performed to determine the correlation between *A. thaliana* and *B. amyloliquefaciens*. We identified 19210 A*. thaliana* genes with RSCU values significantly correlated with *B. amyloliquefaciens* RSCU based on the selection criteria of correlation coefficient (r) >= 0.5 and p-value < 0.05 (considering the median while reducing the probability of false positives and/or false negatives and maintaining significant biological information) (**Additional File 2**). The heatmap showing the frequency of codons for each gene from both species is presented in **Figure 4A**. To investigate the interactions between the proteins corresponding to *A. thaliana* genes with CUB similar to that of *B. amyloliquefaciens*, we chose 2,000 genes with the highest correlation coefficients and constructed the PPI network (**Supplementary Figure S1**), 2,000 being the maximum gene extraction number supported by the string database while ensuring a high correlation.

The network was composed of 448 nodes and 930 edges and had an average number of neighbors of 4.466 (**Figure 4B**). In addition, we ran analysis with the MCODE plugin in Cytoscape to identify hub genes from the PPI network. As a result, we detected 16 clusters, with the highest score hub cluster (cluster1, score = 7.103) containing 30 nodes and 103 edges (**Figure 4B**). These 19210 A*. thaliana* genes were considered to have a similar CUB to that of *B. amyloliquefaciens*, as their RSCU values were positively correlated with those of *B. amyloliquefaciens* (**Additional File 2**). The functional enrichment analysis (**Figure 4C**) revealed that these *A. thaliana* genes were enriched in various biological processes, including response to stress, post-embryonic development, response to abiotic stimulus, response to light stimulus, anatomical structure development, response to chemical, and response to endogenous stimulus, signal transduction, multicellular organism development, and cell communication. The most enriched cellular components were cytoplasm, nucleus, mitochondrion, and plastid, while the most enriched molecular function terms were protein binding, nucleotide binding, DNA-binding transcription factor activity, transcription regulator activity, and DNA binding (**Figure 4C**).

### Role of *A. thaliana* genes with similar CUB to *B. amyloliquefaciens* CUB in high calcium stress adaptability and regulation

**Figure 5:**
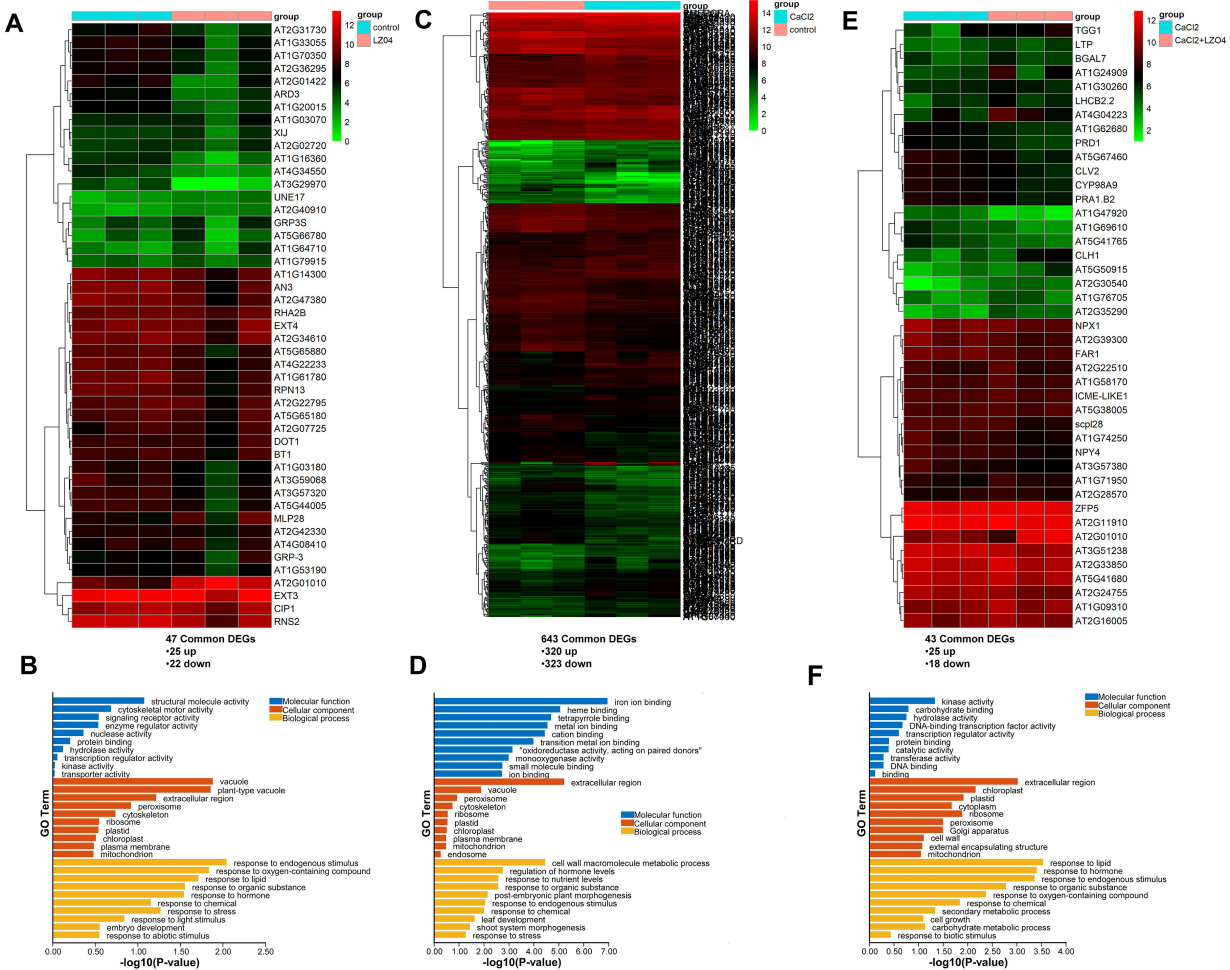
Role of *A. thaliana* genes with similar CUB to *B. amyloliquefaciens* CUB in high calcium stress adaptability and regulation. **(A)** Differential expression analysis of *A. thaliana* Genes with Similar CUB to *B. amyloliquefaciens* CUB between *A. thaliana cultured alone and thaliana cultured in presence of B. amyloliquefaciens under normal condition.* **(B)** Functional enrichment analysis of differentially expressed genes of *A. thaliana* with similar CUB to *B. amyloliquefaciens* CUB between *A. thaliana* cultured alone and thaliana cultured in presence of *B. amyloliquefaciens* under normal condition. **(C)** Differential expression analysis of *A. thaliana* genes with Similar CUB to *B. amyloliquefaciens* CUB between *A. thaliana* cultured alone under calcium stress condition and *A. thaliana* cultured alone under normal condition. **(D)** Functional enrichment analysis of differentially expressed genes of *A. thaliana* with similar CUB to *B. amyloliquefaciens* CUB between *A. thaliana cultured alone under calcium stress condition and A. thaliana cultured alone under normal condition.* **(E)** Differential expression analysis of *A. thaliana* genes with Similar CUB to *B. amyloliquefaciens* CUB between *A. thaliana cultured with B. amyloliquefaciens* under calcium stress condition and *A. thaliana* cultured alone under calcium stress condition. **(F)** Functional enrichment analysis of differentially expressed genes of *A. thaliana* with similar CUB to *B. amyloliquefaciens* CUB between *A. thaliana* cultured with *B. amyloliquefaciens* under calcium stress condition and *A. thaliana* cultured alone under calcium stress condition.

In our previous publication, we studied the role of *B. amyloliquefaciens* LZ04 in the adaptation of *A. thaliana* to high calcium stress (Gong et al., 2021). Results showed that while calcium inhibited growth, *B. amyloliquefaciens* LZ04 improved plant growth under calcium stress conditions (Gong et al., 2021). *A. thaliana* roots grew extensively with *B. amyloliquefaciens* LZ04, and the group had a greater dry weight than the control group (Gong et al., 2021). *B. amyloliquefaciens* LZ04 decreased Na+ content and increased K+ content (Gong et al., 2021). It also attenuated the negative effects of high calcium stress on oxidative stress products and enzyme activities (Gong et al., 2021). Herein, to investigate the potential functions of the *A. thaliana* genes with CUB similar to *B. amyloliquefaciens* genes in these processes, we analyzed the expression of these genes in the *A. thaliana* root under high calcium stress or in combined culture with *B. amyloliquefaciens* LZ04 based on our previously generated transcriptome data. Differential expression analysis using edgeR and DESeq2 packages indicated that the culture in the presence of the *B. amyloliquefaciens* LZ04 strain led to the dysregulation of 47 A*. thaliana* genes with CUB similar to *B. amyloliquefaciens*, with 25 of them being upregulated differentially expressed genes (DEGs) and 22 being downregulated DEGs after merging of upregulated DEGs and downregulated DEGs from both packages (**Additional File 3**). The heatmap in **Figure 5A** indicates the expression profiles of DEGs in *B. amyloliquefaciens* LZO4 vs Control comparison. The DEGs between the two groups were involved in the gene ontology (GO) biological process terms of response to endogenous stimulus, response to oxygen containing compound, response to organic substance, response to hormone, response to chemical, response to abiotic stimulus, response to stress, and response to light stimulus (**Figure 5B**). The most enriched cellular component terms were vacuole, plant-type vacuole, extracellular region, peroxisome, cytoskeleton, ribosome, plastid, chloroplast, plasma membrane and mitochondrion, while the most enriched molecular functions were nucleotide structural molecule activity, cytoskeletal motor activity, signaling receptor activity, enzyme regulator activity, protein binding, and hydrolase activity (**Figure 5B**). Moreover, differential expression analysis indicated that the expression levels of 643 A*. thaliana* genes with CUB similar to *B. amyloliquefaciens* were significantly changed after cultivation of *A. thaliana* under high calcium treatment conditions as compared to the control, and the expression of 323 of these genes was downregulated while the other 320 were upregulated (**Additional File 4**). The heatmap of these significant common DEGs from DESeq2 and edgeR analysis results were as presented in **Figure 5C**. GO analysis indicated the enrichment of the DEGs among both groups in biological process terms of cell wall macromolecule metabolic process, regulation of hormone levels, response to nutrient levels, response to organic substance, post-embryonic plant morphogenesis, response to endogenous stimulus, response to chemical, leaf development, shoot system morphogenesis, and response to stress (**Figure 5D**). The most enriched cellular component terms were vacuole, peroxisome, cytoskeleton, and ribosome, while the molecular function terms such as iron ion binding, heme binding, and tetrapyrrole binding were the most representative (**Figure 5D**). After treating *A. thaliana* cultivated under calcium treatment with the *B. amyloliquefaciens* LZO4 strain, 43 A*. thaliana* genes with CUB similar to *B. amyloliquefaciens* were dysregulated, and 25 of them were upregulated while the other 18 were downregulated DEGs following the merging of upregulated or downregulated DEGs from both differential expression analysis approaches (**Additional File 5**, **Figure 5E**). The DEGs between both groups were associated with the biological process of response to lipid, response to hormone, response to endogenous stimulus, response to organic substance, response to oxygen-containing compound, response to chemical, secondary metabolic process, cell growth, carbohydrate metabolic process, and response to biotic stimulus (**Figure 5F**). The cellular component terms significantly associated with these DEGs were extracellular region, chloroplast, plastid, cytoplasm, and ribosome (**Figure 5F**). In addition, these DEGs were enriched in the molecular function terms of kinase activity, carbohydrate binding, and hydrolase activity (**Figure 5F**).

**Figure 6:**
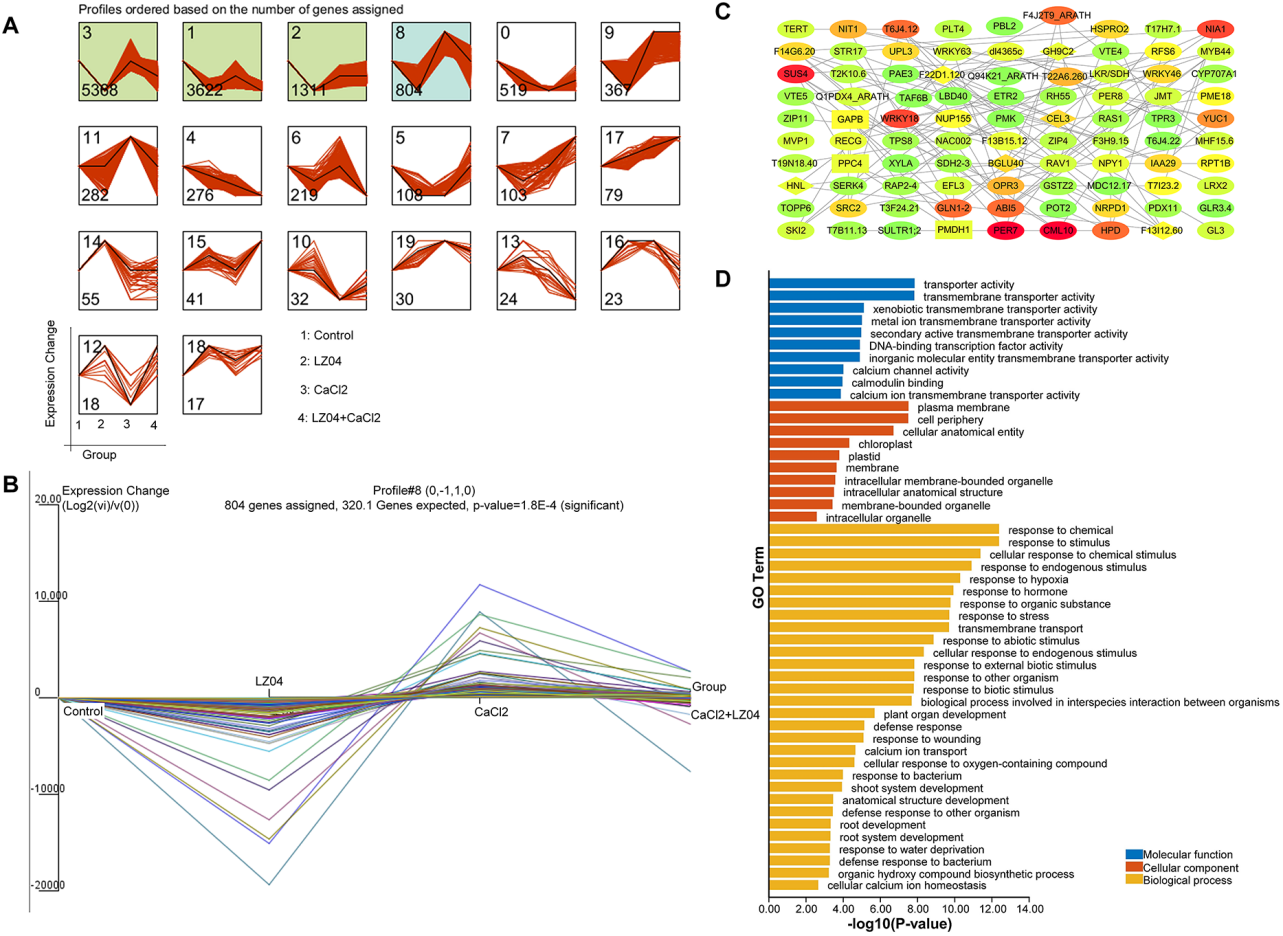
Identification of *A. thaliana* genes with CUB similar to that of *B. amyloliquefaciens* that are involved in the regulation of *A. thaliana* to calcium stress by *B. amyloliquefaciens* by time series clustering. **(A)** Results of time series analysis indicating the expression profiles of the genes. The boxes represent the profiles of the model expressions emerging from STEM analysis. The x-axis shows treatment groups (Control, LZ04, CaCl2, CaCL2+LZ04 in this order), while the y-axis shows the relative value of the normalized expression change against the baseline (Control group). **(B)** Trends in gene expression change in profile 8. **(C)** PPI network of genes in profile 8. Proteins of the same shape belong to the same cluster, while the color indicates the degree obtained from network analysis (higher degrees are represented by red, yellow indicates medium degree, while green represents low degree). **(D)** Functional enrichment analysis of genes in profile 8.

To identify the *B. amyloliquefaciens* LZ04-responsive *A. thaliana* genes with CUB similar to *B. amyloliquefaciens* that was associated with the adaptation of *A. thaliana* to calcium stress, we performed cluster analysis using gene expression profiles (**Figure 6A**). Based on the expression profiles of the *A. thaliana* genes with CUB similar to *B. amyloliquefaciens*, four profiles were identified as significant profiles (P <0.05) (**Figure 6A**) and profile 8 containing 804 genes was identified as *B. amyloliquefaciens*-responsive *A. thaliana* genes with CUB similar to *B. amyloliquefaciens* that was associated with the adaptation of *A. thaliana* to calcium stress (**Figures 6A, B**). The PPI network of genes in profile 8 indicated solid interactions among the proteins corresponding to these genes (**Figure 6C**). The network contained 488 nodes and 760 edges, and the average number of neighbors was 3.467 (**Figure 6C**). MCODE analysis identified Glycoside Hydrolase Family 9C Member 2 (GH9C2), Beta-Glucosidase 40 (BGLU40), Cellulase 3 (CEL3), Hydroxynitrile Lyase (HNL), Beta-Glucosidase 33 (BGLU33), Beta-Glucosidase 30 (BGLU30), Beta-Glucosidase 11 (BGLU11), F22D1.120, A protein of unknown function and F13I12.60 (also a protein of unknown function) as the hub genes assigned to cluster 1 with the highest clustering score (**Figure 6C**). These genes were associated with response to chemical, response to stimulus, cellular response to chemical stimulus, response to endogenous stimulus, response to hypoxia, response to hormone, response to organic substance, response to stress, transmembrane transport, response to abiotic stimulus, cellular response to endogenous stimulus, and response to external biotic stimulus in the GO category of biological process (**Figure 6D**). Terms of biological processes such as response to biotic stimulus, biological process involved in interspecies interaction between organisms, plant organ development, defense response, response to wounding, calcium ion transport, cellular response to oxygen-containing compound, response to bacterium, shoot system development, anatomical structure development, defense response to other organisms, and root development were also enriched. (**Figure 6D**). In the category of cellular component, plasma membrane, cell periphery, cellular anatomical entity, chloroplast, plastid, membrane, intracellular membrane-bounded organelle, intracellular anatomical structure, membrane-bounded organelle, and intracellular organelle were the most enriched terms (**Figure 6D**). In addition, the molecular terms of transporter activity, transmembrane transporter activity, hydrolase activity, DNA-binding transcription factor activity, as well as calcium channel activity, calmodulin binding, and calcium ion transmembrane transporter activity were the most prevalent (**Figure 6D**).

### RT-qPCR validation of gene expression

**Figure 7:**
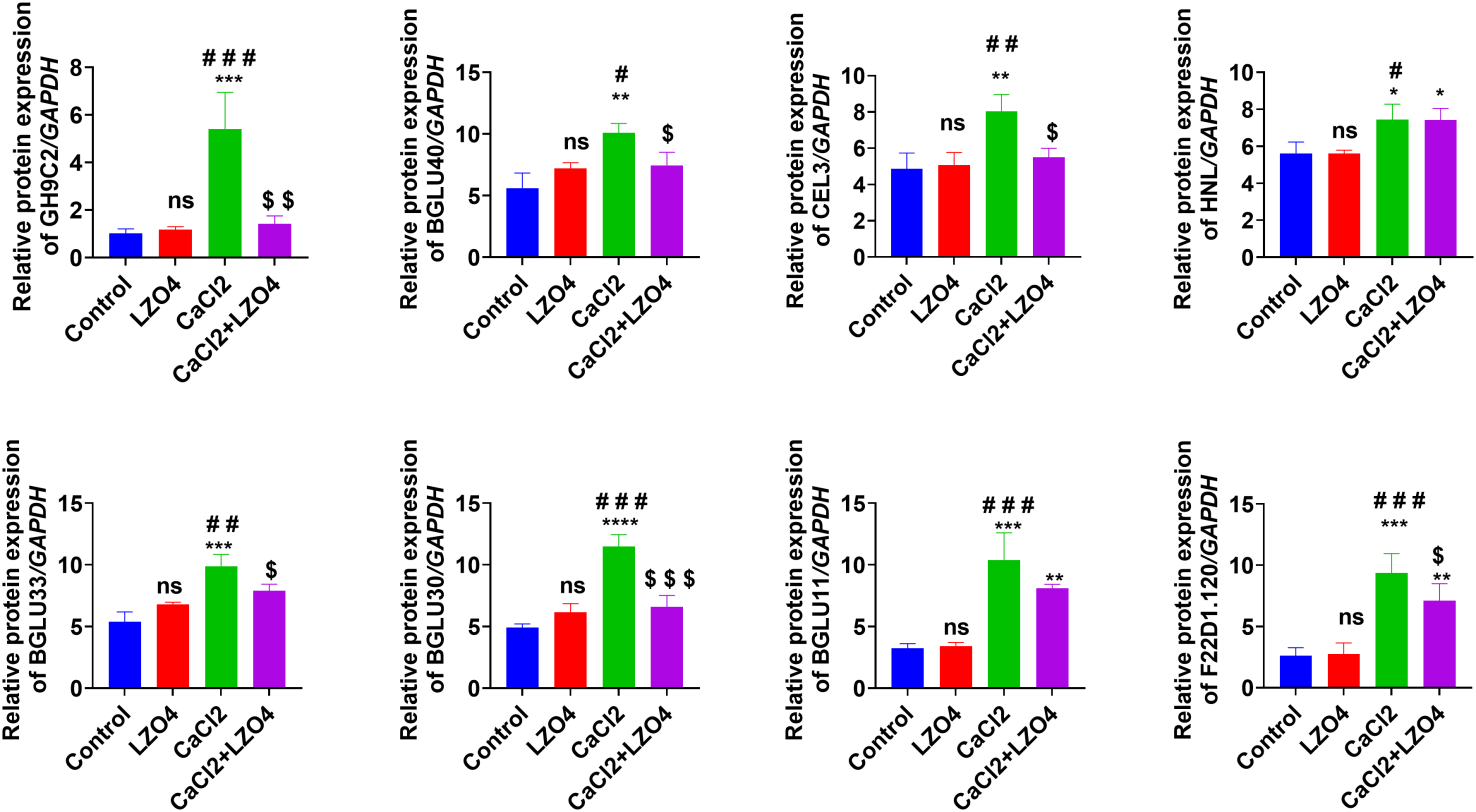
RT-qPCR validation of *A. thaliana genes* with CUB similar to *B. amyloliquefaciens*, involved in calcium stress regulation, identified as hub genes in profile 8 via time series clustering. *p<0.05, **p<0.01, ***p<0.001, and ****p<0.0001 when compared to Control group; #p<0.05, ##p<0.01, and ###p<0.001 when compared to LZ04 group; $p<0.05 and $$$p<0.001 when compared to CaCl2+LZ04 group. ns, no significance.

To confirm the expression levels of the hub genes (GH9C2, BGLU40, CEL3, HNL, BGLU33, BGLU30, BGLU11, and F22D1.120) in profile 8 across the four treatment groups, these genes were selected for RT-qPCR analysis (**Figure 7**). Compared to the Control group, the expression levels of GH9C2, BGLU40, CEL3, HNL, BGLU33, BGLU30, BGLU11, and F22D1.120 were significantly higher in the CaCl2 group, with no notable differences between the LZ04 treatment alone and the Control groups (**Figure 7**). Furthermore, the expression levels of GH9C2, BGLU40, CEL3, BGLU33, BGLU30, and F22D1.120 were significantly decreased in the CaCl2+LZ04 group compared to the CaCl2 group; however, no significant differences in HNL and BGLU11 expression levels were observed between these two groups (**Figure 7**). In general, the patterns of hub gene expression were consistent between the RT-qPCR results and transcriptome sequencing data (**Figure 7**). Additionally, the RT-PCR results indicated that these hub genes play crucial roles in how *A. thaliana* responds to high calcium stress and interacts with *B. amyloliquefaciens* (**Figure 7**).

## Discussion

Calcium is a nutrient and signal molecule that is critical to plant physiology, growth, development, and stress response (Feng et al., 2023). Calcium signals are produced depending on the stimuli of the environment (Ghosh et al., 2022). Exogenous calcium sources can enhance the physiological and biochemical changes of plants, which play the role of protection against different abiotic stresses by activating gene-dependent transcription factors that cause stress tolerance (Ren et al., 2023). For instance, calcium chloride improved drought stress tolerance in barley through an alteration in plant water status, photosynthetic characteristics, antioxidants and osmoprotectant levels, and phytochemicals (Shah et al., 2022). In the same manner, exogenous calcium enhanced growth and photosynthesis capacity rose under drought stress (Zhao et al., 2024). Calcium, likewise, abolished the negative impacts of toxicity with heavy metals affecting growth in plants and boosting antioxidant activities (Abd_Allah et al., 2017; Khalil et al., 2021). It was previously demonstrated that calcium from external sources triggers physiological and biochemical alterations in tree peonies when subjected to drought stress (Zhang et al., 2019b). However, high levels of calcium can induce high calcium stress, greatly affecting the physiology and metabolism of plants. High calcium in the soil will cause plants to take in too much Ca^2+^, which will cause a series of calcium poisoning. The current research has demonstrated that high calcium causes reduced growth, cell and tissue injury, disturbance in nutrient transport and photosynthesis, and stress to some of the crop species (Bachani et al., 2022; Zhao et al., 2024; Zhong et al., 2024). Indeed, high calcium stress can also disturb the phosphate metabolism system and phosphate-based energy metabolism in plants while damaging plant cell membranes, reducing photosynthesis and transpiration rates, and causing leaf senescence; in severe cases, it may even destroy organelles and eventually lead to plant death (Mahajan et al., 2008; Martins et al., 2013). To manage high calcium stress, plants develop signaling pathways with calcium regulators like calcium-dependent protein kinases (CDPKs), cyclic nucleotide-gated ion channels (CNGCs), long non-coding RNAs (lncRNAs), and microRNAs (Li et al., 2020a, 2020; Gong et al., 2021; Yang et al., 2022; Oranab et al., 2023). This results in massive changes in gene expression profiles of essential genes such as transporters, chaperones, and other regulatory genes (Zhang et al., 2019a). Knowledge of these mechanisms is important for the agricultural development of calcium-insensitive crop varieties in karst landforms that are widely distributed, accounting for 12% of the world’s total land area (Wei et al., 2018) and known for their high calcium (with an exchangeable Ca^2+^ content of 2.5-4.3 g·kg-1, which is several times higher than the Ca^2+^ content in conventional soils) and magnesium content, high pH value, and low water storage capacity (Nie et al., 2011; Hao et al., 2015; Geekiyanage et al., 2019).

In this study, we identified 19210 A*. thaliana* genes with CUB similar to that of *B. amyloliquefaciens* and explored their regulatory roles and functions. We found that these genes constitute a strong regulatory network and were involved in the biological processes related to response to stress, post-embryonic development, response to abiotic stimulus, and anatomical structure development. In addition, we found that these genes were involved in the response of *A. thaliana* roots to calcium stress and in the interaction between *A. thaliana* and *B. amyloliquefaciens*. These data suggest that *A. thaliana* genes with CUB similar to *B. amyloliquefaciens* are involved in the interaction of *A. thaliana* with *B. amyloliquefaciens* and can regulate the response of the plant to high calcium stress. Altogether, our study showed, for the first time, the potential role of CUB in mediating plant-PGPR interactions, especially under high calcium stress responses.

CUB plays a significant role in the interaction of the host with their colonizing organism. In previous studies, it was demonstrated that the similarity of the CUB of viruses such as coronaviruses and influenza with that of their hosts influences gene expression dysregulation in the hosts (Nambou and Anakpa, 2020; Nambou et al., 2022). In plants, it was also demonstrated that the similarities between the CUB of plants and that of their colonizing organisms can lead to changes in gene expression in plants (Gupta and Singh, 2021). PGPR plays a significant role in the growth of host plants; this interactive function is driven via various mechanisms involving the production of volatile organic compounds that trigger shifts in plant gene expression and metabolism (Chen et al., 2017; Liu et al., 2017; Gong et al., 2021; Chuang et al., 2022). In our previous studies, we have demonstrated that the *B. amyloliquefaciens* LZ04 interacts with *A. thaliana* and induces significant changes in the lncRNA-miRNA-mRNA regulatory network in this process (Li et al., 2020b). To date, the impact of CUB similarity between *B. amyloliquefaciens* LZ04 and *A. thaliana* has not been studied. The present study revealed for the first time that CUB plays a significant role in the interaction between *B. amyloliquefaciens* LZ04 and *A. thaliana* and in the regulation of the response of *A. thaliana* to calcium stress as well as the beneficial role of *B. amyloliquefaciens* LZ04 in this adaptation process. This study has found important correlations between CUB patterns in *A. thaliana* and *B. amyloliquefaciens*. The correlations suggest that these two organisms share traits that may be the result of co-evolution or selective pressure. These results were corroborated by previous studies demonstrating the relevance of the evolution of *B. subtilis* on plant roots, revealing the role of evolution in fast adaptation and improved root colonization (Nordgaard et al., 2022; Hu et al., 2023; Pomerleau et al., 2024; Richter et al., 2024). Interestingly, it was observed that *A. thaliana* genes with *B. amyloliquefaciens*-like CUB were deregulated when subjected to high calcium stress. However, co-culturing with *B. amyloliquefaciens* reversed these trends. This implies that the codon usage in plants may influence microbial interactions. Stress conditions and microbial factors like metabolites or signaling pathways could alter selective pressures on the plant genome, which could modulate gene expression. *B. amyloliquefaciens* exerted a positive effect, possibly by regulating the stress response pathways or gene expression in the plant. *A. thaliana* genes with CUB similar to *B. amyloliquefaciens* were involved in processes such as response to stress, post-embryonic development, and response to abiotic stimulus. These results suggested that *A. thaliana* tries to keep growing and developing even in tough conditions, and this effect may be partially due to the effect of *B. amyloliquefaciens* on the regulation of stress, growth, and development. The involvement of the response to abiotic stimulus hints that the plant responds to calcium stress in various ways, such as changing ion movement, osmotic balance, and antioxidant production. Thus, our findings revealed complex and diverse stress responses of *A. thaliana* and the potential of *B. amyloliquefaciens* in the regulatory mechanisms. These findings shed light on the mechanisms involved in plant-microbe interactions, with *B. amyloliquefaciens* regulating stress tolerance in *A. thaliana*.

Our study showed a correlation between CUB similarity and *A. thaliana* genes regulating its adaptation to calcium stress, although high gene expression and gene length may be potential confounding factors. In highly upregulated genes, translational selection may cause a significant codon bias, and hence, increase the correlation between these genes and the bacterial CUB. Also, short genes exhibit lower codon variability due to the low number of codons they contain, which could introduce errors in the calculations of CUB. Thus, during CUB calculations, it is necessary to normalize for gene length and control for gene expression levels. Moreover, though the high correlation of *A. thaliana* genes with *B. amyloliquefaciens* genes in CUB is interesting and corresponds to the pathways known to regulate stress, it does not necessarily imply a direct functional connection. Indeed, factors such as gene expression level, GC content, and tRNA abundance can also significantly contribute to CUB, with overexpressed genes exhibiting stronger CUB; this can amplify correlation without necessarily being relevant to function (Dos Reis et al., 2003; Jia and Li, 2005; Liu et al., 2022; Parvathy et al., 2022). Moreover, the shared usage patterns of the same gene within regulatory or co-expression modules imply that enrichments of high-CUB-similarity genes may indicate co-expression clustering but not necessarily mechanistic interaction (Najafabadi et al., 2009). As a matter of fact, genome-wide results have demonstrated that codon usage is correlated with expression trends, rather than absolute expression levels, which indicates the risk of misinterpreting the effect of false positives as significant in terms of functionality (Najafabadi et al., 2009; Parvathy et al., 2022).

Despite the fact that the majority of previous studies on PGPR have not specifically focused on CUB, a number of works have conveyed a comparable trend in physiological and transcriptional patterns between dicots and monocots. As an example, in *A. thaliana* and other dicots, PGPR treatments generally induce stress-responsive pathways which include ROS signaling, ion transport, hormone signaling, and carbohydrate metabolism pathways (Chiapello et al., 1998; Kawabe and Miyashita, 2003; Jia and Xue, 2009; Cardinale et al., 2013; Singha et al., 2014; Camiolo et al., 2015; Mazumdar et al., 2017; Das and Bansal, 2019; Galicia-Campos et al., 2024), which are regulated by *A. thaliana* genes with CUB similar to that of *A. amyloliquefaciens* genes as shown in our research. Although the limitations of classical analyses are the pattern of expression or the cascade of regulation, our study brings additional knowledge through demonstrating the convergence at the codon level between host and microbe. Rice (Oryza sativa) or maize codons are featured by a marked difference in codon usage compared with dicots; monocots are somewhat biased toward codons with a GC ending, particularly in highly expressed genes whereas eudicots (including *A. thaliana*) exhibit both types of biases, A/T and G/C ending codons (Kawabe and Miyashita, 2003; Jia and Xue, 2009; Cardinale et al., 2013). Such variations imply that the convergence of codon usage with PGP, such as *B. amyloliquefaciens* (a GC-rich bacterium), can be dissimilar in different lineages of plants. In *A. thaliana*, housekeeping and photosynthesis genes at high levels of expression are G/C biased and stress-responsive or tissue-specific genes are A/T biased (Chiapello et al., 1998). Translational selection has been inferred under the stress conditions in rice because there are strong correlations between the usage of C/G ending codons and expression level of genes (r > 0.8) (Jia and Xue, 2009; Cardinale et al., 2013). One codon-specific study of stress-induced MAPK genes across *A. thaliana*, soybean (Glycine max), and rice found that codon usage patterns varied by species and stress type, emphasizing that both mutational bias and translational selection play roles in shaping codon usage under stress (Singha et al., 2014). In this case, although the other studies involving PGPR do not include a measure of codon bias, they are still consistent in showing that PGPR increases resilience by modulating genes related to transport, metabolism, defense, and homeostasis (functions in our convergent genes on CUB). The effects associated with codon usage convergence can, however, be dependent on the codon context of the host. *A. thaliana* (a moderate GC-content, moderate codon bias organism) could be matched with a GC-biased bacterium more easily than a monocot with an extreme preference for codons ending in G and C. Thus, there are patterns specific to dicot systems that need to be tested in monocots or other dicot lineages.

Yet, our study has some limitations. First of all, we focused only on the interaction between *A. thaliana* and *B. amyloliquefaciens*, while exploring the role of CUB in the interaction among other bacterial species and plants could provide a wider understanding. Secondly, we did not investigate the role of post-transcriptional regulation or conduct metabolomic, physiological, or functional validation of genes with CUB similar to that of *B. amyloliquefaciens*. A Further expression analysis is therefore needed to clarify the role of these genes in adaptation to calcium stress or plant-bacteria interaction. Thirdly, this study does not test the molecular mechanisms linking CUB to translational efficiency or recognition between species. It would also be interesting to study how mutants of these *A. thaliana* genes, which have a CUB similar to that of *B. amyloliquefaciens*, respond under calcium stress conditions alone or in combination with *B. amyloliquefaciens* treatment; we plan to conduct this analysis independently in future studies. Finally, studying the involvement of CUB in the response of *A. thaliana* to other types of stress could provide a more complete understanding of its role.

## Conclusions

This study analyzed the impact of CUB similarity between *A. thaliana* and *B. amyloliquefaciens* on their interaction, especially under high-calcium stress conditions. Our results show that *A. thaliana* genes displaying CUB patterns similar to those of *B. amyloliquefaciens* can significantly impact the resilience of the plant through regulating processes such as ion transport, carbohydrate metabolism, chemical response, and cellular homeostasis. These results shed light on the molecular mechanisms beneath the adaptation of *A. thaliana* to environments with elevated calcium content and emphasize the regulatory impact of *B. amyloliquefaciens* in this context. This work lays the groundwork for identifying potential gene-editing targets and developing customized, plant-specific synthetic microbiomes. Ultimately, these insights may inform innovative strategies to enhance *A. thaliana* growth and productivity in calcium-rich or karst soils.

## Data Availability

The RNA-seq data have been deposited into the CNGB Sequence Archive (CNSA) of China National GeneBank DataBase (CNGBdb) with accession numbers CNP0000745 and CNP0000640.

## Supplementary material

Supplementary Figure 1 Protein-protein interaction network of *A. thaliana* genes with CUB similar to the CUB of *B. amyloliquefaciens* coding sequences.

Additional File 1 CUB data of *B. amyloliquefaciens* sequences.

Additional File 2 A*. thaliana* genes with CUB similar to the CUB of *B. amyloliquefaciens* coding sequences.

Additional File 3 Differential expression analysis of *A. thaliana* genes with CUB similar to the CUB of *B. amyloliquefaciens* coding sequences between *A. thaliana* alone and *A. thaliana* cultured in presence of *B. amyloliquefaciens* LZO4 under normal cultivation conditions.

Additional File 4 Differential expression analysis of *A. thaliana* genes with CUB similar to the CUB of *B. amyloliquefaciens* coding sequences between *A. thaliana* alone and *A. thaliana* cultured under calcium stress conditions.

Additional File 5 Differential expression analysis of *A. thaliana* genes with CUB similar to the CUB of *B. amyloliquefaciens* coding sequences between *A. thaliana* cultured in the presence of *B. amyloliquefaciens* LZO4 under calcium stress conditions and *A. thaliana* alone under calcium stress conditions.
