# Supplementary figures and images for: Arabidopsis thaliana genes with codon usage bias similar to that of B. amyloliquefaciens are involved in the regulation of A. thaliana adaptation to high calcium stress by B. amyloliquefaciens
Source: Front Plant Sci. 2025 Sep 1;16:1623360. doi: 10.3389/fpls.2025.1623360 (PMC12446992; doi:10.3389/fpls.2025.1623360)

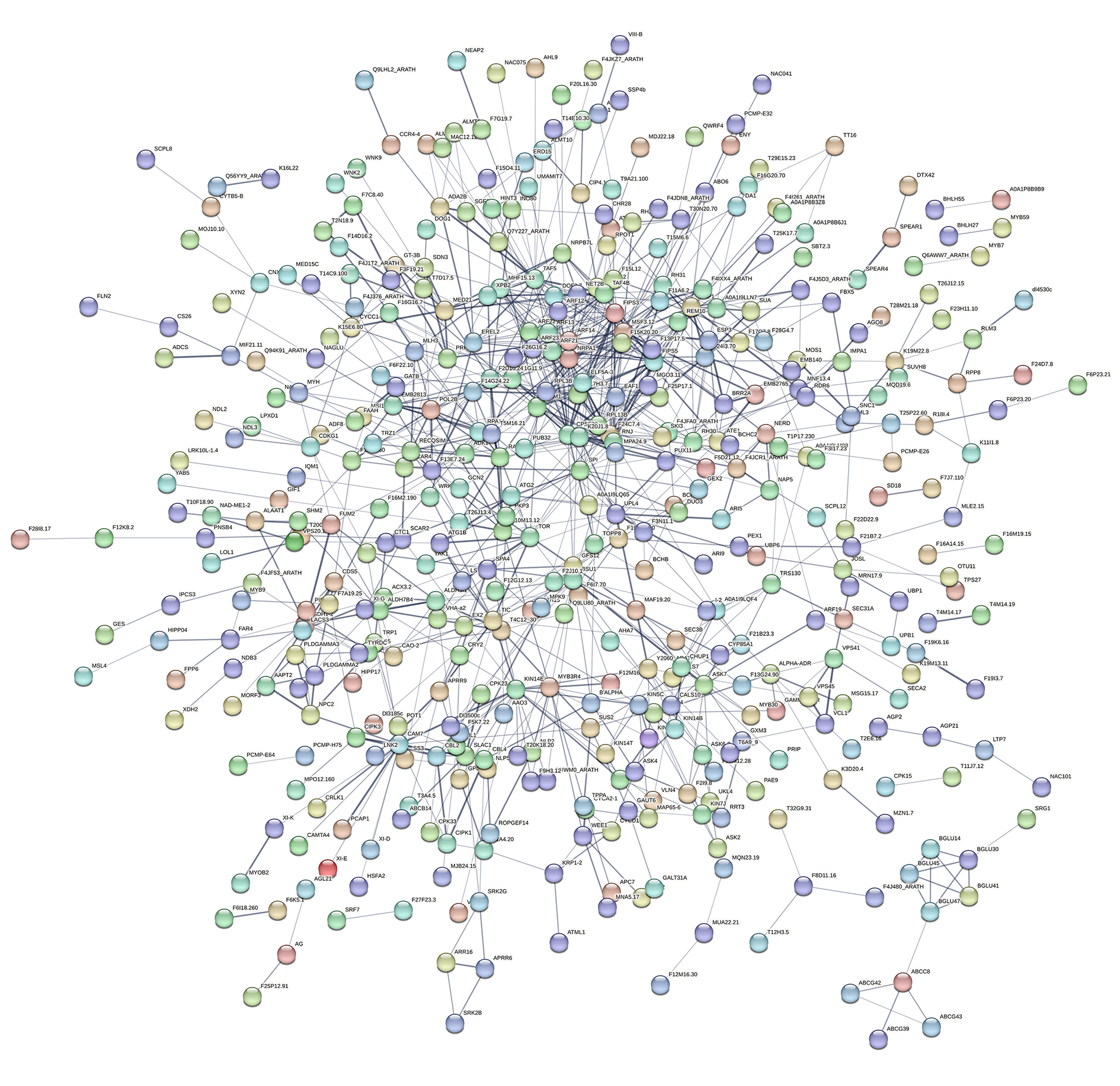

Supplement: Supplementary Figure 1 — Protein-protein interaction network of A. thaliana genes with CUB similar to the CUB of B. amyloliquefaciens coding sequences. [file Image1.tif]
