# Supplementary material for: Arabidopsis thaliana genes with codon usage bias similar to that of B. amyloliquefaciens are involved in the regulation of A. thaliana adaptation to high calcium stress by B. amyloliquefaciens
Source: Front Plant Sci. 2025 Sep 1;16:1623360. doi: 10.3389/fpls.2025.1623360 (PMC12446992; doi:10.3389/fpls.2025.1623360)
Supplement: Supplementary file 7 [file Table1.docx]

**Supplementary Table S1: Primers used in RT-qPCR validation of key genes**

| **Gene** | **Forward (5’→3’)** | **Reverse (5’→3’)** |
| --- | --- | --- |
| GH9C2 | ATCTTGCCGGAGAAACAGCA | GGCGTGAGTGAGTAGTAGCC |
| BGLU40 | AGGCAGTTTTCCAAAGGGCT | GTAGGACCTCTGCCTTCTGC |
| CEL3 | TCACACGCACTTCGTTCTCA | GACCTCCTACGATTGCTCCG |
| HNL | ATACAACAGCCGACCGAGG | AGGTTCATATACGTTACGCTGTT |
| BGLU33 | CCCCAAAATGTCCCACAGGA | CACCCTCTTGGCACTTTTTACA |
| BGLU30 | TTCCCTAACCAGTTCAACAAACT | TGTACCGTCGTCGTTGTCATT |
| BGLU11 | ATGGATTAGCATAAGAGCTGGAT | ACGAAAGCGTATGTCTTGCT |
| F22D1.120 | TAGTGAAACTGTGGAAGAAGCAA | AGCAGCAGCCACGATACAG |
